# Supplementary material for: Acetylcholine is released in the basolateral amygdala in response to predictors of reward and enhances the learning of cue-reward contingency
Source: eLife. 2020 Sep 18;9:e57335. doi: 10.7554/eLife.57335 (PMC7529459; doi:10.7554/eLife.57335)
Supplement: Transparent reporting form [file elife-57335-transrepform.pdf]

Westbrook Centre, Milton Road
Cambridge CB4 1YG
UK

P 01223 855340
W elifesciences.org
T @elife

eLife's transparent reporting form

We encourage authors to provide detailed information *within their submission* to facilitate the interpretation and replication of experiments. Authors can upload supporting documentation to indicate the use of appropriate reporting guidelines for health-related research (see EQUATOR Network), life science research (see the BioSharing Information Resource), or the ARRIVE guidelines for reporting work involving animal research. Where applicable, authors should refer to any relevant reporting standards documents in this form.

If you have any questions, please consult our Journal Policies and/or contact us: editorial@elifesciences.org.

Sample-size estimation

- You should state whether an appropriate sample size was computed when the study was being designed
- You should state the statistical method of sample size computation and any required assumptions
- If no explicit power analysis was used, you should describe how you decided what sample (replicate) size (number) to use

Please outline where this information can be found within the submission (e.g., sections or figure legends), or explain why this information doesn't apply to your submission:

We computed the required sample size for a 90% power level with an alpha of 0.05 by estimating the control (EYFP) group mean would be 10 rewards and the mean experimental (ChR2) group would be 20 rewards with a standard deviation of 5. We utilized a power calculator for continuous outcomes of two independent samples, assuming a normal distribution. The result was 6 samples per group. Each manipulation experiment started with at least 6 mice were included in each group (this information is found in the Methods section).

Replicates

- You should report how often each experiment was performed
- You should include a definition of biological versus technical replication
- The data obtained should be provided and sufficient information should be provided to indicate the number of independent biological and/or technical replicates
- If you encountered any outliers, you should describe how these were handled
- Criteria for exclusion/inclusion of data should be clearly stated
- High-throughput sequence data should be uploaded before submission, with a private link for reviewers provided (these are available from both GEO and ArrayExpress)

Please outline where this information can be found within the submission (e.g., sections or figure legends), or explain why this information doesn't apply to your submission:

eLife Sciences Publications, Ltd is a limited liability non-profit non-stock corporation incorporated in the State of Delaware, USA, with company number 5030732, and is registered in the UK with company number FC030576 and branch number BR015634 at the address Westbrook Centre, Milton Road Cambridge CB4 1YG, UK | March 2019

In each experiment, each animal within a group served as a biological replicate. These studies did not include technical replicates, however the behavioral paradigm was repeated multiple times and baseline learning curves were highly reproducible across all replicates, increasing confidence that manipulations resulted in specific changes to the learning curves. Similarly, the fiber photometry studies using different methods of evaluating ACh levels and activity of ACh terminals yielded highly similar outcomes, validating the conclusions of these studies. Each main fiber photometry experiment was carried out in two independent cohorts of mice.

Mice were only excluded from analyses if fluorescence was not observed at injection sites, if a behavioral chamber malfunctioned (e.g. syringe pump failed), or if they received the improper compound. No outliers were excluded. Fiber photometry mice were excluded from analyses if they did not meet the acquisition criterion by the last day of Training (this information is found in the Methods section).

Statistical reporting

- Statistical analysis methods should be described and justified
- Raw data should be presented in figures whenever informative to do so (typically when N per group is less than 10)
- For each experiment, you should identify the statistical tests used, exact values of N, definitions of center, methods of multiple test correction, and dispersion and precision measures (e.g., mean, median, SD, SEM, confidence intervals; and, for the major substantive results, a measure of effect size (e.g., Pearson's r, Cohen's d)
- Report exact p-values wherever possible alongside the summary statistics and 95% confidence intervals. These should be reported for all key questions and not only when the p-value is less than 0.05.

Please outline where this information can be found within the submission (e.g., sections or figure legends), or explain why this information doesn't apply to your submission:

When comparing acquisition of the cue-reward learning task, differences between groups and interactions across days for Pre-Training, Training, Extinction data were evaluated using Two-Way Repeated Measures ANOVAs. Raw data for each individual mouse undergoing behavioral manipulations (optogenetic and pharmacological) are shown in the corresponding supplemental figures. Heatmaps for fiber photometry experiments show individual mouse and cohort averaged data in the main and supplemental figures. In the fiber photometry experiments, boostrapped 99% confidence intervals were constructed to determine when fluorescent signal was significantly different from 0 (more details in Methods section and figure legends). Additional repeated measures analyses (Real Time Place Preference and locomotion) were performed using Two-Way Repeated Measures ANOVAs while discrete tests (Light-Dark Box) were analyzed with an unpaired t-test. Behavioral summary line and bar graphs display mean and SEM and/or individual data (this information is found in the Methods section and in figure legends). Fiber photometry signal line graphs display 99% confidence intervals with the trial level mean overlaid, except when comparing reference and signal channels, during which mean and SEM are displayed, with all information listed in figure legends.

eLife Sciences Publications, Ltd is a limited liability non-profit non-stock corporation incorporated in the State of Delaware, USA, with company number 5030732, and is registered in the UK with company number FC030576 and branch number BR015634 at the address Westbrook Centre, Milton Road Cambridge CB4 1YG, UK | March 2019

(For large datasets, or papers with a very large number of statistical tests, you may upload a single table file with tests, Ns, etc., with reference to sections in the manuscript.)

Group allocation

- Indicate how samples were allocated into experimental groups (in the case of clinical studies, please specify allocation to treatment method); if randomization was used, please also state if restricted randomization was applied
- Indicate if masking was used during group allocation, data collection and/or data analysis

Please outline where this information can be found within the submission (e.g., sections or figure legends), or explain why this information doesn't apply to your submission:

Mice were randomly assigned to EYFP or ChR2 groups, controlling for average group age. Mice were randomly assigned to antagonist group. Each mouse was pseudo-randomly assigned to behavioral chamber when multiple chambers were used, counterbalancing for groups across boxes (this information is found in the Methods section). Masking was not applied during data acquisition but data analyses were semi-automated in MATLAB and performed blind to condition.

Additional data files ("source data")

- We encourage you to upload relevant additional data files, such as numerical data that are represented as a graph in a figure, or as a summary table
- Where provided, these should be in the most useful format, and they can be uploaded as "Source data" files linked to a main figure or table
- Include model definition files including the full list of parameters used
- Include code used for data analysis (e.g., R, MatLab)
- Avoid stating that data files are "available upon request"

Please indicate the figures or tables for which source data files have been provided:

All source data files and code have been uploaded to Dryad.

eLife Sciences Publications, Ltd is a limited liability non-profit non-stock corporation incorporated in the State of Delaware, USA, with company number 5030732, and is registered in the UK with company number FC030576 and branch number BR015634 at the address Westbrook Centre, Milton Road Cambridge CB4 1YG, UK | March 2019
